# Introduction to the Special Issue: Application of Essential Oils in Food Systems

**DOI:** 10.3390/foods7040056

**Published:** 2018-04-05

**Authors:** Juana Fernández-López, Manuel Viuda-Martos

**Affiliations:** IPOA Research Group (UMH-1 and REVIV-Generalitat Valenciana), Department of AgroFood Technology, Escuela Politécnica Superior de Orihuela, Miguel Hernández University, Ctra. Beniel km. 3,2, E-03312 Orihuela, Alicante, Spain; j.fernandez@umh.es

**Keywords:** essential oil, foods, preservatives

## Abstract

Essential oils have received increasing attention as natural additives for the shelf-life extension of food products due to the risk in using synthetic preservatives. Synthetic additives can reduce food spoilage, but the present generation is very health conscious and believes in natural products rather than synthetic ones due to their potential toxicity and other concerns. Therefore, one of the major emerging technologies is the extraction of essential oils from several plant organs and their application to foods. Essential oils are a good source of several bioactive compounds, which possess antioxidative and antimicrobial properties, so their use can be very useful to extend shelf-life in food products. Although essential oils have been shown to be promising alternative to chemical preservatives, they present special limitations that must be solved before their application in food systems. Low water solubility, high volatility, and strong odor are the main properties that make it difficult for food applications. Recent advances that refer to new forms of application to avoid these problems are currently under study. Their application into packaging materials and coated films but also directly into the food matrix as emulsions, nanoemulsions, and coating are some of their new applications among others.

## Introduction

**Figure 1:**
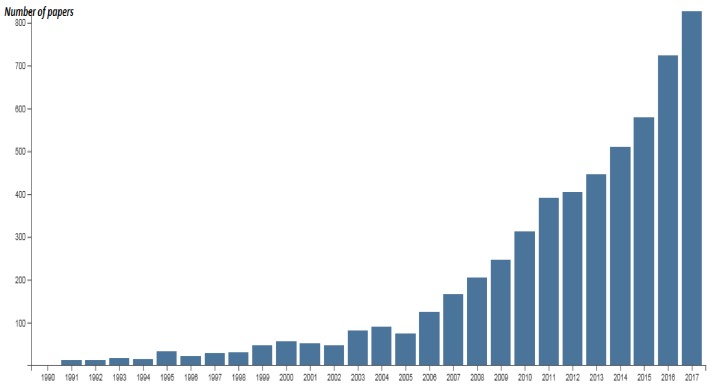
Evolution of the number of papers per year (from 1990 to 2017) published regarding essential oils and foods.

The application of essential oils (EOs) in food systems as natural inhibitors or biopreservatives has received increasing attention mainly due to consumer concerns toward chemical preservatives. This increasing interest can be checked by the number of papers published related to EOs application in foods. In a basic search using the Web of Science database, from 1950 to 2017, selecting as search topic “essential oils and foods” and as document type “article” a total of 5559 results were obtained. Although the first article found dates from 1953 [1] and since then a trickle of articles appear in the following years, it is not until 1990 that articles published every year are reported. Figure 1 shows the evolution of the number of papers per year (from 1990 to 2017) published regarding essential oils and foods. As can be seen in this figure, more than 86% of these papers have been published in last decade which reveals the currently of the topic addressed in this special issue.

The application of essential oils for shelf-life extension in foods is mainly due to their antioxidant and antimicrobial properties which also is reflected in the number of papers found when the words “antioxidant” (1920 papers), “antimicrobial” (2473 papers), or both (973 papers) were added as searching criterion. Regarding the type of foods mainly used in these studies, it can be concluded that essential oils have been applied as biopreservatives in all types of foods, although their application in fruits and vegetables has been the highest reported: fruits (657 papers), vegetables (403 papers), fish products (415 papers), meat products (410 papers), milk and dairy products (216 papers), and bread and baked foods (97 papers).

One of the most important aspects that has changed along time is the way in which essential oil has been applied in foods. The first method of application was directly added essential oil to the food matrix, which showed special limitations mainly associated with intrinsic obstacles such as their low water solubility, high volatility, low stability, bioavailability, and strong odor. EOs are unstable volatile compounds which can be degraded easily (by oxidation, volatilization, heating, light, etc.) when they are added to the food matrix. It must be taken into account that most of the food elaboration processes include heat treatment or air and light exposition, all of them factors that increase their degradation. For these reasons, several protection methods to increase their action duration and to provide a controlled release during the shelf-life of food have been proposed. Encapsulation has emerged as a useful alternative to enhance EO stability. The encapsulation of EOs using different materials and methods has been widely studied [2]. EOs have been encapsulated in polymeric particles, liposomes, and solid lipid nanoparticles, which enhanced its stability and efficacy. The recent advances in nanotechnology have made possible the development of novel carrier agents for the delivery and control release of EOs in food system with enhanced chemical, oxidative, and thermal stability [3]. Although nanoencapsulation is a promising tool for effective delivery of EOs into food, the toxicological aspects of most of the nanocarriers and their molecular target site must be further explored.

EOs have also been used as additives in biodegrabable films and coatings for active food packaging [4,5]. EOs can provide the films and coatings with antioxidant and/or antimicrobial properties, depending both on their composition and on the interactions with the polymer matrix. The antioxidant activity depends not only on the specific antioxidant activity of the oil compounds but also on the film’s oxygen permeability. The incorporation into edible films can promote the antimicrobial capacity of EOs, and the effectiveness of the edible film against microbial growth will depend on the oil’s nature and the type of microorganism. EOs’ controlled release from edible films is another aspect that positively affects their effectiveness. In addition, consumers’ concern regarding possible negative health effects of applying synthetic preservatives to food products together with the boom of organic culture that promotes the consumption of organic foods (in whose processing synthetic additives are not authorized) have also contribute to boost the interest in organic EOs properties.

In this special issue, the original papers published address all these aspects providing further insights into the application of EOs in foods or assessing specific properties relevant in a specific type of EO.

In the study by Pellegrini et al. [6] the authors focused their interest in EOs from some officinal plants from the Abruzzo territory (Italy), assessing the whole chemical characterization of their volatile fraction and their antimicrobial and antioxidant activities. All these analyses allow establishing which of them could be better candidates for their potential application as biopreservatives depending on the type of food to be incorporated. In addition, the characterization of EOs autochthonous from a specific region will allow their application by local industries contributing to their development.

The study by Sharopov et al. [7] also contributes to increasing the knowledge of local EOs, in this case it is regarding the EO of Fennel from Tajikistan. These authors assessed the chemical composition of this EO by gas chromatographic-mass spectrometric analysis and its antioxidant activity. In addition, these authors also studied the potential cytotoxic activity against several cancer cell lines, which is very important for its potential application in food products.

Consistent with this aspect there is also the paper of Satyal et al. [8] contributing to a deeper knowledge about EOs from a culinary ingredient broadly used around the world (garlic). In this case, EOs from both, garlic (*Allium sativum*) and other type of garlic widely used as its substitute (*Allium vineale*), have been chemically characterized.

Ballester-Costa et al. [9] have focused their study in EOs from four Thymus species from organic growth, contributing to their potential application to organic food processing. Taking into account that thymus is a common specie in the Spain meat industry, the work has assessed its antioxidant and antibacterial properties with the objective of its use for the meat industry. The main novelty of this work is the application, as culture medium for the antibacterial activity evaluation, and of several meat homogenates (minced beef, cooked ham, or dry-cured sausage). This type of study allows that the potential effect of these meat matrices on bacterial survival would be included in the general antibacterial activity that has been evaluated.

In the work carried out by Nowotarska et al. [10] the antimicrobial modes of action of six compounds from cinnamon and oregano EOs against *Mycobacteriun avium* sbsp. *paratuberculosis* were evaluated. It is a pathogenic bacterium that can infect food animals and humans and to be present in milk, cheese, and meat which reveals the interest in studying some compounds to be inhibited and their action mechanisms. 

Others two papers are related to the effect of thyme and savory EOs on specific foods: Santoro et al. [11] reported the effect on the control of postharvest diseases and quality of peaches and nectarines, while Banani et al. [12] reported its efficacy on apples. In the first work the authors concluded that both EOs favor a reduction of brown rot incidence (caused by *Monilinia fructicola*) but increased gray mold (caused by *Botrytis cinerea*). Respect to the overall quality of the fruits, both EOs showed a positive effect in reducing weight loos and in maintaining ascorbic acid and carotenoid content. Regarding the second study, apples treated with these EOs showed lower gray mold severity and incidence and the authors reported that the PR-8 gene of apple may play a key role in the mechanism implicated in this inhibition.

The prevention of growth of *Escherichia coli* in ground beef by the application of surfactant micelle-entrapped eugenol was the objective proposed in the paper by Tolen et al. [13]. In this case the authors concluded that this antimicrobial treatment did not significantly decontaminate ground beef and so further studies must be proposed to increase the utility of these EOs for beef safety protection.

Application of essential oils in food systems is an interesting and growing area for researchers whose results could end up having a great use for food industries. It is a wide field of research where different aspects can be addressed. We hope that readers will find this issue interesting and useful and allow them to understand its importance and relevance and so to propose new studies for future papers.
